# Seroprevalence of Nipah virus and related paramyxoviruses in native frugivorous bats, Luzon, Philippines

**DOI:** 10.1080/22221751.2025.2555720

**Published:** 2025-09-29

**Authors:** Marana S. Rekedal, Mary-Glazel Noroña, Jairue Azel P. Café, Neil Mittal, Sophie A. Borthwick, Kirk J. Taray, Jezryl Jaeger L. Garcia, Samantha L. Magsanoc, Hazel R. Cruz, Dorothy Jane Manzano, Lianying Yan, Dolyce H. W. Low, David T. S. Hayman, Mary Grace Dacuma, Catalino Demetria, Phillip A. Alviola, Fedelino F. Malbas, Gavin J. D. Smith, Eric D. Laing

**Affiliations:** a Department of Microbiology and Immunology, Uniformed Services University of Health Sciences, Bethesda, MD, USA; b Henry M. Jackson Foundation for the Advancement of Military Medicine, Rockville, MD, USA; c Research Institute for Tropical Medicine, Muntinlupa, Philippines; d Programme in Emerging Infectious Diseases, Duke-NUS Medical School, Singapore; e University of the Philippines Los Baños, Laguna, Philippines; f Massey University, Palmerston North, New Zealand

**Keywords:** Nipah virus, bats, Philippines, biosurveillance, spill over risk

## Abstract

Nipah virus (NiV) is a highly virulent zoonotic virus classified as a priority pathogen and biohazard. In 2014, an outbreak of NiV-like disease in the Province of Sultan Kudarat, Mindanao, Philippines resulted in a 53% case fatality rate. Here, we identified wildlife bat hosts of NiV by conducting monthly serological surveillance of flying foxes and other native frugivorous bat species across Luzon. We estimated 13.92% NiV seroprevalence in native flying foxes. We also detected NiV neutralizing activity in some flying fox sera and identified factors such as age and seasonality as drivers of high anti-NiV antibody levels. In contrast, less than 10% NiV seroprevalence was detected in *R. amplexicaudatus*, *C. luzoniensis*, and *P. jagori* bats, and these bats have no detectable neutralizing antibodies. This is the first serological description of NiV in native flying foxes in the Philippines, highlighting a major wildlife host in an understudied region.

## Introduction

Zoonotic viruses including the priority pathogen, Nipah virus (NiV) (genus *Henipavirus*, family *Paramyxoviridae*) pose significant epidemic and pandemic potential. NiV first emerged in Peninsular Malaysia in 1998 when infected pigs transmitted the virus to pig farmers and abattoir workers; the export of infected pigs resulted in spread to Singapore [1,2]. Though pigs served as the intermediate, amplifying host for the first NiV outbreak, subsequent outbreaks of the bat-borne virus have had differing routes of spillover from wildlife into human populations. Annual NiV outbreaks in Bangladesh since 2001 are due to direct wildlife to human transmission, linked to the consumption of unpasteurized date palm sap contaminated with secreted or excreted virus by flying foxes [3,4]. Since 2018, annual outbreaks of NiV disease have been recorded in Kerala, India, although the interface(s) and routes of transmission remain uncharacterized [5–7].

In 2014, an outbreak of NiV-like henipaviral disease occurred in two villages (Tinalon and Midtungok) approximately 15 km apart in the Province of Sultan Kudarat, Mindanao, Philippines [8]. Human cases were primarily linked to contact with and consumption of sick horses, reminiscent of outbreaks of the close relative of NiV, Hendra virus (HeV), in Australia [8,9]. The recovered partial phosphoprotein gene sequence closely aligned with 99% nucleotide identity to NiV isolates from the Malaysia outbreak, suggesting NiV as the infecting henipavirus rather than HeV [8]. This outbreak resulted in 17 cases and nine deaths and occurred from March-May 2014, with five of the 17 cases traced to human-to-human virus transmission [8]. The variety in NiV spillover paths across outbreaks in Malaysia, Bangladesh, and the Philippines highlights the importance of location-specific biosurveillance to accurately assess regional risk.

Flying foxes belonging to the *Pteropus* genus are the confirmed reservoir for henipaviruses NiV, HeV, and Cedar virus (CedV) [10,11]. Over 30 years of biosurveillance efforts in Australia have described the ecological role of native flying foxes in promoting HeV circulation and maintenance, which has been critical in spatiotemporal spillover risk predictions [12–14]. Wildlife surveillance in Thailand, Cambodia, Malaysia, India, and Bangladesh have detected serological and molecular evidence of NiV in multiple flying fox species [15–25]; however, to date, the wildlife source of the 2014 outbreak in the Philippines has not been identified. In the absence of approved medical countermeasures for NiV disease, the development of early warning systems and epidemiological interventions aimed at mitigating spillover remain critical.

To reduce the biothreat risk of NiV, it is critical to identify the wildlife hosts from which NiV is likely to emerge and characterize the timing of NiV transmission from these hosts in understudied areas. The objectives of this study are to identify the major wildlife bat host(s) for NiV and identify patterns of NiV transmission within the reservoir host(s), which can be used to develop spatiotemporal models of emergence risk. We utilized wildlife sero-surveillance of several fruit bat species, including flying foxes, sampled on a monthly basis at multiple locations on the Luzon island of the Philippines. We hypothesized that we would identify evidence of NiV in native species of flying foxes belonging to the *Pteropus* and *Acerodon* genera.

## Methods

### Serum sample collection

Bats sampled include flying foxes (*Pteropus hypomelanus*, *Pteropus vampyrus*, and *Acerodon jubatus*), Geoffroy's rousette bats (*Rousettus amplexicaudatus)*, dawn bats (*Eonycteris robusta* and *Eonycteris spelaea*)*,* Peters's fruit bats (*Cynopterus luzoniensis*), and greater musky fruit bats (*Ptenochirus jagori*). Species identification, along with sex and age determination, were performed by wildlife experts based on bat physical appearance, with age categorization for neonate, juvenile, sub-adult, young adult, and adult bats as previously defined [26,27]. All species were sampled monthly from July 2023 to August 2024, excluding August 2023, in the municipalities of Agno, Doña Remedios Trinidad (DRT), Tayabas, Burdeos, and Infanta on Luzon. Venous blood was collected from the cephalic vein on the wing of the bat sampled and centrifuged at 4,000 RPM for 10–15 min. Collected sera supernatant ranged from 10 µL to 3 mL and was diluted 1:10 in phosphate buffer solution (PBS) and stored at −80°C.

### Multiplex microsphere-based immunoassay (MMIA)

Serological measurements were conducted using an antigen-based multiplex microsphere immunoassay (MMIA) built with Multi-Analyte Profile (xMAP) technology (Luminex; Austin, TX, USA). Henipaviral antigens include NiV (Accession Number: NC_002728.1), HeV (NC_001906.3), CedV (NC_025351.1), Ghana virus (GhV) (NC_025256.1), and Mòjiāng virus (MojV) (NC_025352.1) receptor-attachment surface glycoproteins (G), and bat-borne pararubulavirus antigens include Sosuga virus (SosV) (YP_009094033.1), Yeppoon virus (YepV), and Grove virus (GroV) hemagglutinin-neuraminidases (HN). Protein sequences of the HN of novel pararubulaviruses, YepV and GroV, were provided by Dr. Ina Smith and are available upon request. Expression of envelope attachment receptor-binding G and HN antigens in native-like quaternary conformations and procedures for coupling to magnetic microspheres (MagPlex, Luminex) for multiplex serology have been detailed previously [28–32]. A mock protein antigen was purified from stable HEK cell lines transfected with an empty expression plasmid, and affinity column supernatant was quantified and coupled to a unique microsphere region.

A detailed MMIA protocol has been previously documented [30,32,33]. Major differences from cited protocols and reagents used are explicitly noted here. Thawed serum samples were thermally inactivated (60°C for 30 min) for biosafety handling [34]. Serum samples were diluted 1:100 and further to 1:500 in PBS (Fischer Scientific 21040CMX12) to allow for two replicates of each sample. Antigen-microsphere coated plates was incubated sequentially with (1) diluted serum samples, (2) biotinylated-protein A and G (Thermo Fischer 29989 and 29988) diluted 1:1000 in PBS-Tween 20 (PBST) for secondary detection, and (3) streptavidin-R-pycoerythrin (Strep-PE, Fischer Scientific S866) diluted 1:1000 in PBST to detect antigen–antibody complexes. Antigen–antibody complexes were resuspended in PBST, measured by a MAGPIX multiplexing machine (Luminex), and reported as median fluorescence intensity (MFI) units of a minimum of 50 microspheres per unique region.

### NiV surrogate virus neutralisation test (sVNT)

A subset of available serum samples collected from flying foxes, *R. amplexicaudatus*, *C. luzoniensis,* and *P. jagori* bats were tested for neutralizing capacity using a receptor-blocking antigen-based MMIA surrogate virus neutralization test (sVNT) [35]. To identify MFI thresholds where neutralizing capacity would be likely observed, we selected flying fox samples with anti-NiV-G binding antibody MFI levels that ranged from 500–1000 (Bin 1), 1000–3000 (Bin 2), and > 3000 MFI (Bin 3). *R. amplexicaudatus*, *C. luzoniensis*, and *P. jagori* bat samples were selected at similar anti-NiV-G binding MFI ranges. Most serum samples were diluted 1:20, 1:40, 1:80, and 1:160 in PBS, with some serum samples also diluted 1:10 and 1:320, and applied to the same multiplex of paramyxovirus antigen-coupled microspheres. Instead of biotinylated-protein A and G, we used biotinylated-ephrin B2 (Fischer Scientific BT496), a receptor for NiV, HeV, CedV, and GhV [11,36,37], at 125 ng/mL [35]. Antigen–antibody and antigen-receptor complexes were resuspended in PBST and measured on a MAGPIX machine, with lower MFI values indicating neutralizing activity. Anti-NiV monoclonal antibody (mAb) m102.4 [38] control was tested at concentrations of 10.0, 5.0, 1.0, 0.5, 0.1, 0.05, and 0.01 µg/mL to provide a reference for a proper sigmodal inhibition curve expected to indicate NiV neutralizing activity. The mAb was used only as a plate positive control and was not used as a reference for interpolation.

NiV neutralization capacity was assessed by half-maximal inhibitory concentration (IC_50_) values, which were reported as the inverse of the dilution factor at which 50% neutralization occurs. As tested dilutions ranged from 1:10 to 1:320, we assumed best estimates for maximum IC_50_ = 320 and minimum IC_50_ = 10. Any bat serum samples with MFI results from the sVNT that did not form four-parameter inhibition (4PL) curves were considered to not have detectable NiV receptor-blocking neutralizing activity.

### Data analysis

All sample MFI results had a plate-specific PBS background MFI subtracted to account for any signal due to the resuspension solution. Any samples with inconsistent results across 1:100 and 1:500 dilutions were retested to increase technical accuracy and minimize any effects of error. Serology data were sorted into bat species groups of flying foxes (*P. hypomelanus, P. vampyrus,* any unidentified *Pteropus* species, and *A. jubatus*), *R. amplexicaudatus* bats, *Eonycteris* species (*E. spp.*; *robusta* and *spelaea*) bats, *C. luzoniensis* bats, and *P. jagori* bats. To extrapolate sero-reactivity profiles within each group, dimensionality of paramyxovirus serology data was reduced using principal component analysis (PCA), retaining six components optimized to include at least >70% of the variance. This analysis was applied to scaled MFI results collected from both 1:100 and 1:500 sera dilutions and no significant difference was observed; further analysis and presentation was completed with results collected from the 1:100 sera dilution.

Univariate mixture model analysis was utilized to generate MFI cutoffs for seropositivity, with distinct mixture model analysis performed for every identified virus antigen-bat species combination. We also accounted for possible MFI noise using the mock MFI values of each bat species group to represent species-specific assay noise of random antibody binding antigen. The threshold for noise cutoff was the species mock MFI value at 95% specificity. Therefore, we performed mixture model analysis both with MFI values within the species-specific assay noise and with the removal of these MFI values. Seropositivity cutoffs were determined at 90% sensitivity of both two- and three-populations model to estimate our seronegative and seropositive populations. All statistical analyses were performed using the statistical software R v4.4.3 (R Core Team, 2024) [39]. Key packages were *stats* v3.6.2 (2019) for PCA, *factoextra* v1.0.7 (2020) for PCA and K-medoids clustering [40], *mixtools* v2.0 (2022) for mixture model analysis [41], and *INLA* v4.4 (2025) for prevalence estimations [42,43].

## Results

### Identification of sero-reactive profiles

**Figure 1.**
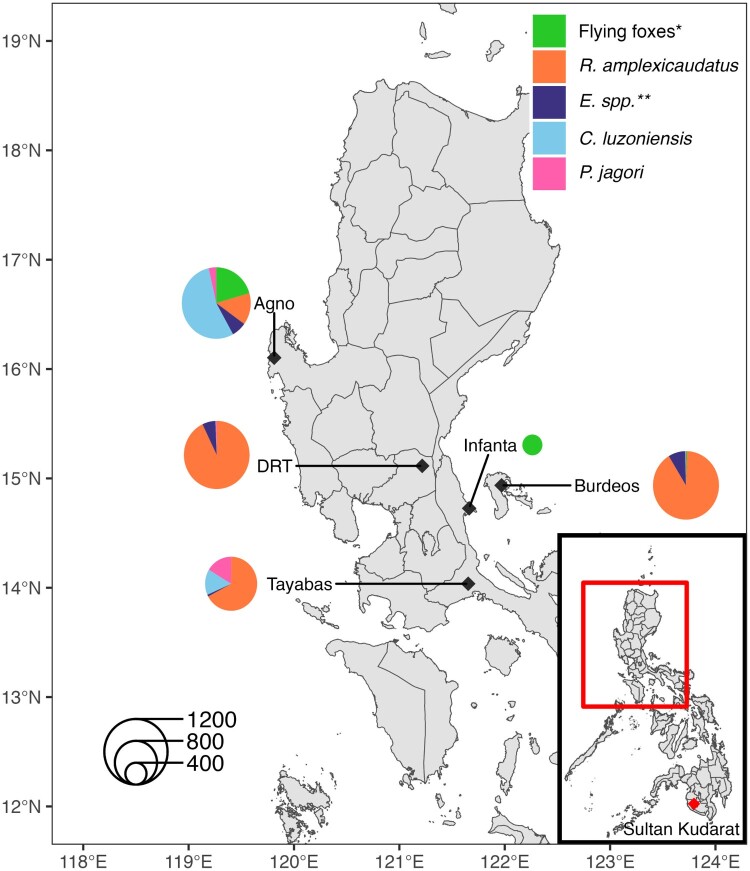
Sampling sites and bat collection breakdown. Location of sampling sites within Luzon island, Philippines and the proportion of each sampled bat species at each location. Sites include Infanta, Agno, Doña Remedios Trinidad (DRT), Burdeos, and Tayabas. Species collected include flying foxes*, *Rousettus amplexicaudatus*, *Eonycteris* species (E. spp.**), *Cynopterus luzoniensis*, and *Ptenochirus jagori*. Red diamond indicates the location of the 2014 NiV-like outbreak in Sultan Kudarat province. *Flying foxes include *Pteropus vampyrus, Pteropus hypomelanus, Acerodon jubatus* bat species; **E. Spp. include *Eonycteris spelaea* and *robusta* bat species

**Figure 2.**
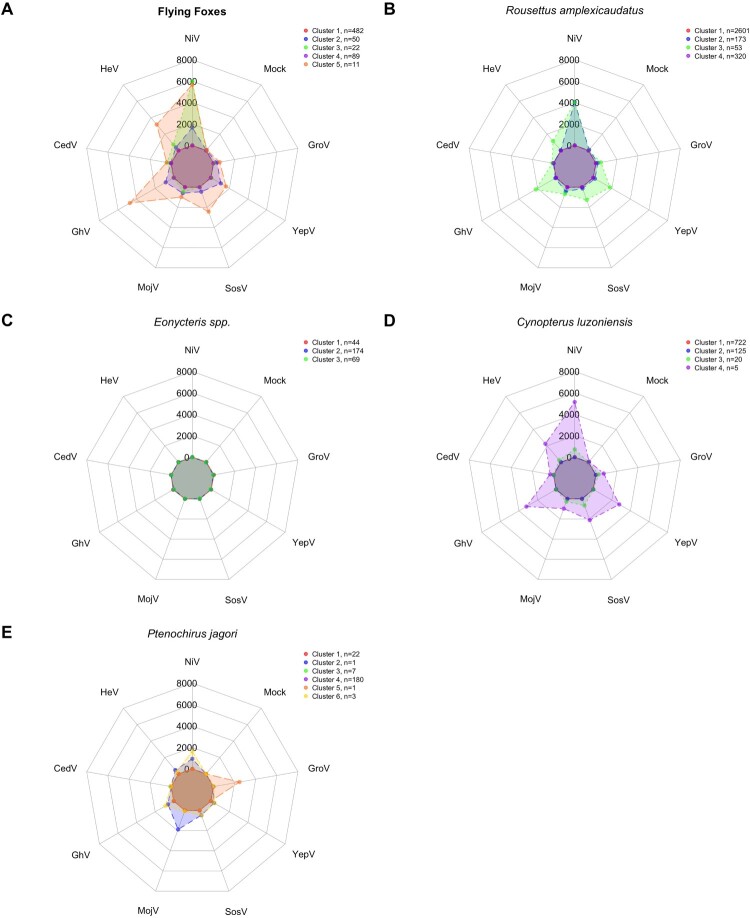
Sero-reactivity profiles of multiple bat species groups for paramyxoviruses. Radarcharts demonstrating sero-reactivity profiles to eight paramyxovirus G and HN antigens and a mock control protein for Philippine frugivorous bats, including (A) flying foxes (*P. vampyrus, P. hypomelanus, A. jubatus*; N = 654), (B) *R. amplexicaudatus*; N = 3147, (C) *E.*species (*E. spelaea* & *E. robusta*; N = 287), (D) *C. luzoniensis* (N = 872), and (E) *P. jagori* (N = 214) during monthly collections on Luzon island from July 2023 – August 2024, excluding August 2023. Radial axes represent each of the eight antigens used as the serological target for detection of antibodies. Scales are a continuous linear measurement of median fluorescence intensity (MFI) from 0 – 6000 that represent antibody levels. Connecting lines represent the individual mean values for each clusters based on k – medoids clustering after PCA to six components where cumulative explained variance is >70%.

A total of 5174 bats were sampled from July 2023 to August 2024, including 654 flying foxes (*P. hypomelanus*, *P. vampyrus*, and *A. jubatus*), 3147 *R. amplexicaudatus* bats, 287 *Eonycteris* species (*E. spp.*) bats, 872 *C. luzoniensis* bats, and 214 *P. jagori* bats across all sites at varying distributions (Figure 1, STable 1). The sero-reactivity profiles of each bat species were examined using PCA to identify major virus-host relationships (SFigure 1, S Figure 2, and STable 2).

In flying foxes, five clusters were identified; Cluster 2 and 3 have distinct sero-reactivity to NiV, and Cluster 5 has simultaneous sero-reactivity to multiple henipaviruses (NiV, HeV, and GhV) and, to a lesser extent, distantly related pararubulaviruses (SosV and YepV) (Figure 2A). In *R. amplexicaudatus* bats, four clusters were identified; Cluster 2 has sero-reactivity to primarily NiV and Cluster 3 has simultaneous sero-reactivity to NiV and HeV, and GhV, SosV, and YepV (Figure 2B). In the *E. spp*. of bats, three clusters were identified; however, sero-reactivity was at similar levels to the mock control (Figure 2C). In *C. luzoniensis* bats, four clusters were identified; Cluster 4 has simultaneous sero-reactivity to NiV and HeV, and GhV, SosV, and YepV, similar to the cluster observed in both flying foxes and *R. amplexicaudatus* (Figure 2D). In *P. jagori* bats, six clusters were identified; Cluster 6 has simultaneous sero-reactivity to henipaviruses NiV and GhV (Figure 2E). Though Cluster 2 has simultaneous sero-reactivity to MojV and NiV and Cluster 5 has distinct sero-reactivity to GroV, each cluster is only made up of a single bat (Figure 2E).

**Figure 3.**
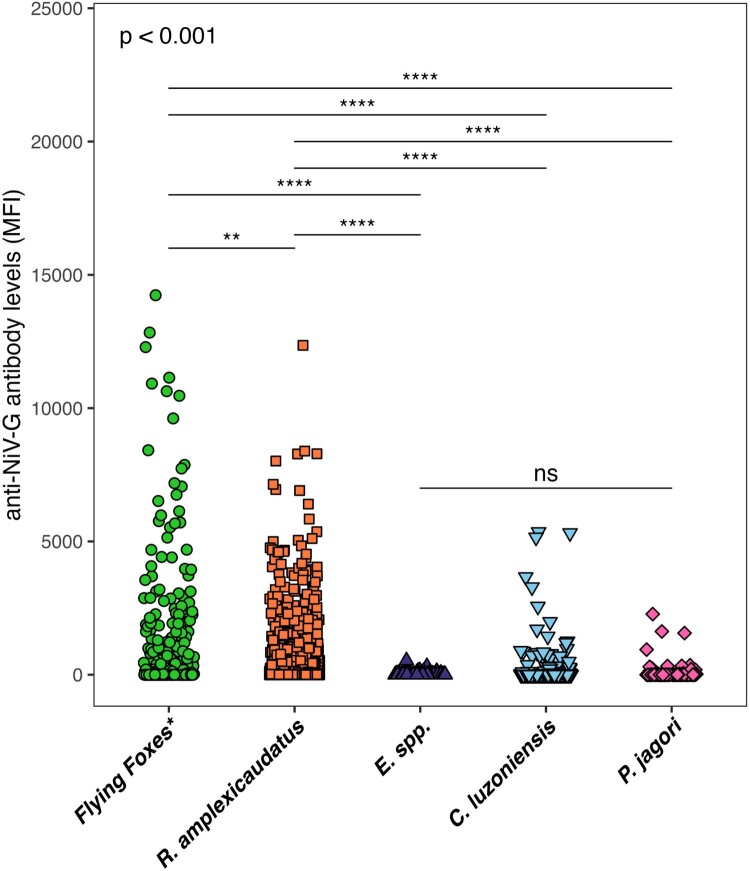
Comparison of anti-NiV-G antibody response among all bat species groups. Comparisons of anti-NiV-G antibody levels among flying foxes (green circle), *R. amplexicaudatus* bats (orange square), *Eonycteris* species bats (purple up triangle), *C. luzoniensis* bats (blue down triangle), and *P. jagori* bats (pink diamond) using a Kruskal – Wallis test (*p*<0.001). Multiple comparisons by Dunn’s test indicated that any comparisons among *E. spp*, *C. luzoniensis*, and *P. jagori* bats were non – significant. Comparison of flying foxes and *R. amplexicaudatus* was significant (*p*<0.01) and multiple comparisons of flying foxes and *R. amplexicaudatus* to any other species was significant (*p*<0.0001).

Although both flying foxes and *R. amplexicaudatus* shared a distinctly NiV sero-reactive cluster (Figure 2A,B), the overall magnitude of detectable antibodies against NiV was higher in flying foxes (*p* < 0.01) (Figure 3). NiV sero-reactivity observed in *E. spp., C. luzoniensis*, and *P. jagori* bats were significantly much weaker than the other bat groups (*p* < 0.0001) (Figure 3). Although trends were observed among these three bat species, differences did not reach statistical significance (Figure 3). Cluster 5 in flying foxes (*n* = 11/654)*,* Cluster 3 of *R. amplexicaudatus* bats (*n* = 53/3147), and Cluster 4 of *C. luzoniensis* bats (*n* = 5/872) formed a similar shape with sero-reactivity to both henipaviruses and multiple pararubulaviruses (Figure 2A, B, & D).

### Neutralization capacity for NiV

We next examined NiV sera receptor-blocking surrogate neutralization capacity of the NiV sero-reactive bats. We selected 70 serum samples from adult flying foxes and 12 serum samples from juvenile/subadult flying foxes. Of the flying fox serum samples tested, 14.6% (12/82) had detectable NiV receptor-blocking activity (Table 1; SFigure 3A-B). All 12 serum samples with neutralizing capacity had anti-NiV-G binding antibody levels > 3000 MFI, suggesting the detection of circulating neutralizing antibodies is more likely in serum samples with higher antibody magnitudes (SFigure 3A). In contrast, *R. amplexicaudatus*, *C. luzoniensis*, and *P. jagori* bat sera samples had no detectable neutralizing activity, even at comparable anti-NiV-G binding antibody levels > 3000 MFI (SFigure 3C-D; SFigure 4). An anti-NiV mAb provided a plate positive control for 4PL inhibition curve fitting (SFigure 3E).

**Table 1.** Demographic factors and IC50 values for flying foxes with detectable NiV neutralization activity.

Sample	Month Collect	Location	Species	Sex	Age*	BMI (g/m2)	NiV binding (MFI)	IC50 (1/dilution factor)
00000329	Jul 2023	Burdeos	*P. hypomelanus*	F	Ad	2.16	10634	320
00000417	Jul 2023	Infanta	*P. hypomelanus*	M	Ad	3.62	5710	54
00000431	Jul 2023	Infanta	*P. hypomelanus.*	M	Ad	3.75	12834	62
00002822	Feb 2024	Infanta	*P. hypomelanus*	F	Ad	2.99	7737	60
00003324	Mar 2024	Infanta	*P. vampyrus*	F	Juv	2.40	6752	60
00003762	Apr 2024	Infanta	*P. hypomelanus*	F	Ad	2.83	11142	60
00003767	Apr 2024	Infanta	*P. vampyrus*	M	Ad	4.44	5979	60
00004403	May 2024	Infanta	*P. hypomelanus*	M	Ad	3.42	10464	320
00004416	May 2024	Infanta	*P. hypomelanus*	M	Ad	2.89	3972	33
00005039	Jul 2024	Infanta	*P. hypomelanus*	F	Ad	2.03	9614	34
00005055	Jul 2024	Infanta	*P. vampyrus*	M	SbAd	1.77	3192	12
00005142	Jul 2024	Agno	*P. hypomelanus*	F	Ad	3.15	14235	26

*Ages categories are subadults (SbAd), juveniles (Juv), and adults (Ad).

### NiV seroprevalence estimates

To estimate NiV seroprevalence, we performed two population univariate mixture model analysis, assuming that the sampled bats contain seronegative and seropositive sub-populations. Based on density plots of anti-NiV-G binding MFI levels, we selected appropriate seropositivity cutoffs at 90% sensitivity in a two population model (SFigure 5A-D; STable 3). When analysis was performed without the removal of assay noise, we observed that cutoffs were much lower, with *R. amplexicaudatus* NiV cutoff within its species-specific assay noise MFI threshold, highlighting the importance of removing assay noise to appropriately guide population distributions (SFigure 5A-D; STable 3). We also explored NiV seropositive cutoffs in a three sub-populations model, accounting for a possible intermediate group and potentially calculating a more conservative seropositivity cutoff (SFigure 6). The output of cutoffs were similar to our two population calculated cutoffs, resulting in an intermediate group that may be challenging to interpret (STable 4). Thus, we selected the two population mixture model analysis given the underlying assumption of a binary population. Final NiV seropositivity cutoff in flying foxes with a two population mixture model was 904 MFI (90% sensitivity, 93% specificity), resulting in an estimated NiV seroprevalence of 13.92 (95% CI [13.50–13.77])% in flying foxes (Table 2). In contrast, NiV seroprevalence was less than 10% for the other bat species, being 6.20 (6.15–6.27), 2.87 (2.85–2.91), and 1.40 (1.40–1.44)% seroprevalence for *R. amplexicaudatus*, *C. luzoniensis*, and *P. jagori* bats, respectively (Table 2).

**Table 2.** NiV seroprevalence estimates in different bat species groups with selected seropositivity cutoff.

Bat Type	Total N	Seropositivity cutoff* (MFI, % specificity)	Seropositive (N)	NiV seroprevalence (95% CI)
Flying foxes (*P. hypomelanus*, *P. vampyrus*, *A. jubatus*)	654	904 (93)	89	13.61 (13.50 - 13.77)
*R. amplexicaudatus*	3147	984 (93)	195	6.20 (6.15 - 6.27)
*C. luzoniensis*	872	455 (97)	25	2.87 (2.85 - 2.91)
*P. jagori*	214	947 (100)	3	1.40 (1.40 - 1.44)

*Cutoffs are determined at 90% sensitivity

### NiV seroprevalence drivers

**Figure 4.**
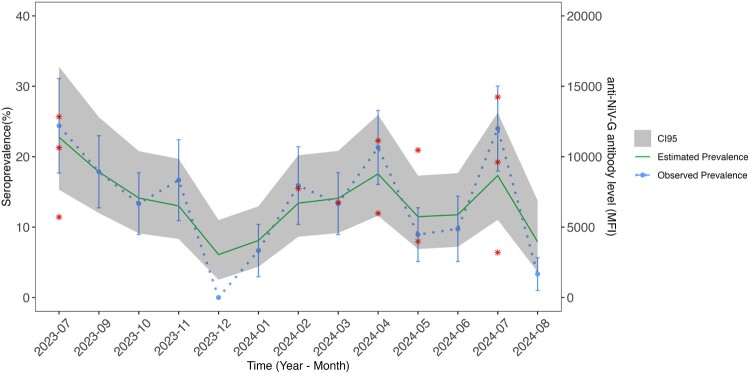
Seasonal differences in NiV seroprevalence and occurrence of neutralizing activity in flying foxes. Monthly changes in NiV seroprevalence in flying foxes during the period of sampling from July 2023 to August 2024, excluding August 2023. Observed seroprevalence (blue circle, dotted line) was calculated using cutoff = 904 MFI for NiV seropositivity with lower and upper 95% CI indicated, estimated seroprevalence is based on Bayesian smoothing function, and 95% CI (grey shaded) were calculated for each month. Red stars mark the flying fox samples that had NiV neutralizing activity at their respective anti-NiV-G antibody levels (MFI).

As NiV seroprevalence was highest in flying foxes, we assessed the associated demographic factors. First, we examined roost location and observed a significant difference in NiV serological response between flying foxes sampled from the Infanta and Agno sites (*p* < 0.05), though the maximum magnitude of the anti-NiV-G response was comparable (SFigure 7A). Second, we examined differences between species and identified that *P. hypomelanus* had higher anti-NiV-G antibody levels compared to *P. vampyrus* and *A. jubatus* bats (*p* < 0.05) (SFigure 7B). For age, we observed significant difference in anti-NiV-G antibody response between juvenile and adult flying foxes (*p* < 0.0001) (SFigure 7C), no significant sex differences in anti-NiV-G antibody response (SFigure 7D), and significant difference between pregnant and not pregnant female bats (*p* < 0.05) (SFigure 7E). Lastly, we modelled NiV seroprevalence over one year and observed a peak starting in the months of February-May and through July when NiV neutralization was also detected (Figure 4).

## Discussion

We identified *Pteropus* and *Acerodon* flying foxes as the most likely NiV wildlife host in the Philippines, consistent with its reservoir status across South and Southeast Asia. *P. vampyrus* and *P. hypomelanus* flying foxes are distributed in several South and Southeast Asian countries, and molecular and serological evidence of NiV infection has been found in these species [16,21]. *A. jubatus* flying foxes are found only in the Philippines; sero-surveillance has suggested its potential as a host for *Reston ebolavirus* in the Philippines by ELISA [44], but no studies have examined its host capacity for henipaviruses, particularly NiV. As *A. jubatus* is closely related to the *Pteropus* genus (previously considered *P. jubatus*), it is unsurprising that all three species are likely NiV hosts in comparison to other examined frugivorous bat species.

Examining NiV neutralizing activity of selected serum samples allowed us to improve specificity for NiV response over HeV, and to explore the responses for NiV in non-flying fox species. Furthermore, we were also able to verify the threshold cutoff utilized for seropositivity with an effector-function assay. All neutralizing activity was detected in flying fox serum samples with anti-NiV-G binding antibody levels within the > 3,000 MFI bin. These serum samples are all above the second cutoff (1993 MFI) in the three population mixture model analysis and these data may help refine future research diagnostics cutoffs (STable 4). However, this threshold cutoff was less sensitive than the one generated in the two sub-population model that we utilized for the seroprevalence estimate. Understanding the biological implications of the differences in the threshold cutoffs generated by the two and three populations mixture models may assist in refining future interpretations of intermediate classes within populations, for example, sub-populations that have recovered from infection and have detectable binding antibodies, but for which neutralizing antibodies have waned. These class identifications may be a beneficial subpopulation for inclusion in future models that are focused on time-post-infection dynamics.

Ultimately, our results indicate flying foxes with relatively high anti-NiV-G binding antibody level as the likeliest group for detection of NiV neutralization. Assuming detectable antibody responses decay within three months [45], combined detection of high antibody levels and neutralizing antibodies likely indicates recent infection. The sVNT is specific for detecting neutralizing antibodies that block receptor binding by competing for binding to the receptor epitope pocket of NiV-G [35], and offers some insight into antibody affinities. Other known neutralizing antibodies bind outside of the pocket [38], and thus this receptor-blocking sVNT is limited in quantifying the total neutralization capacity of the detectable sera antibodies. Age-related factors could be playing a role, as most with neutralizing activity were adults, further suggestive that higher affinity receptor-blocking neutralizing antibodies are a factor of multiple NiV infections through the individual bat’s life history (Table 1). However, the small sample size limits our ability to make conclusions about underlying immunological factors and processes for detecting NiV neutralization.

We detected NiV sero-reactivity in *R. amplexicaudatus*, *C. luzoniensis*, and *P. jagori* bats, suggesting virus-host relationships; however, the lack of observed NiV neutralization capacity despite anti-NiV antibody levels comparable to flying foxes suggest that (i) it is not likely NiV that is infecting these species, (ii) we may be detecting serological response for a NiV-like henipaviruses, and/or (iii) these bat species may not be major NiV hosts. These bats may play a role as secondary hosts in the Philippines, but native flying foxes remain the most relevant host for NiV. There is precedent for NiV seropositivity and molecular detection of NiV RNA in *R. leschenaultii* bats in India, Vietnam, and China [46–49]; in Vietnam, *R. leschenaultii* bat species also had weak evidence of neutralization for NiV [48]. In contrast, experimental infection of *R. aegypticus* bats were found to not sustain productive NiV infection, with no detectable virus in tissues and no evidence of seroconversion [50].

It has been suggested that *Rousettus* bats do not make productive neutralizing antibody responses, even against viruses that they are known hosts for [45,51,52]. This could explain our contrasting results of NiV sero-reactivity in *R. amplexicaudatus* bats, but no detectable NiV neutralizing activity. Further, both *C. luzoniensis* and *P. jagori* bat species with NiV sero-reactivity did not demonstrate NiV neutralization, though our sample size inhibits our ability to fully describe it. Some seropositivity has been observed in *C. sphinx* species in Vietnam for NiV nucleoprotein (NP) target, which may lend less specificity to NiV and be more indicative of a NiV-like henipavirus [48]. Studies in Thailand with *C. sphinx* did not find any NiV seropositivity [53]. In Malaysia, *C. brachyotis* and *E. spelaea* bats were found to have NiV seropositivity with weak neutralization [19]. Based on our results, further examination will be needed to understand these bat species’ role(s) in NiV transmission chains. Recent detection of novel henipaviruses in *Rousettus leschenaultii* sampled in southern China hints that our detection of anti-NiV G binding antibodies that do not outcompete ephrin B2 for the G receptor-binding pocket is consistent with the circulation of antigenically-distinct novel NiV-like henipaviruses in the non-flying fox species [54].

We observed simultaneous henipavirus and pararubulavirus sero-reactivity in flying foxes and *C. luzoniensis* bats (Figure 2). GhV sero-reactivity does not occur independently, suggesting presence of common ancestral viruses that are causing simultaneous sero-reactive profiles for both GhV and NiV, since NiV-G immunization causes limited cross-reactive serological response with GhV-G [55]. Further research is needed to understand whether this observation is due to a genus (*Henipavirus*) or family (*Paramyxoviridae*) level association. No distinct profile for a single pararubulavirus was detected in flying foxes and *C. luzoniensis* bats (Figure 2A, D). We did not calculate seroprevalence for other viruses because the sero-reactivity profiles did not indicate the presence of one, single known virus, unlike the observed NiV sero-reactivity.

The observed seasonal differences in NiV seroprevalence and neutralization in flying foxes suggest peak NiV transmission in February–March through July (Figure 4). To date, we are unable to draw strong conclusions about the demographic factors that may be influencing seasonality of NiV transmission*.* Furthermore, the study is limited in that it provides a cross-sectional snapshot, precluding assessment of infection dynamics, antibody waning, or reinfection. Future studies over a longer period of time will be necessary to detect nucleic acid evidence of NiV, and ultimately to construct a comprehensive, predictive mechanistic model for insight into specific processes and populations that drive NiV transmission.

The earliest horse cases in the 2014 NiV – like outbreak were traced to March 3rd, with additional horse cases detected as late as May 11th [8]. The overlap of peak NiV transmission in sampled flying foxes with the known outbreak highlights February–March as a possible temporal spillover risk. Our sampling efforts on Luzon focused on describing spillover risk near the Metro Manila area, the most populous area within the Philippines, though the known outbreak occurred in the southern island of Mindanao. Additional sero-surveillance will be needed to validate our initial observed temporal trends and assess spillover risk internal and external of the Metro Manila area. Our results indicate that further research is necessary to understand spatiotemporal spillover risk of henipaviruses, underlying drivers of NiV transmission in flying foxes, and NiV transmission chains from wildlife to livestock toward pre-pandemic preparedness efforts in an understudied region.

## Supplementary Material

Supplemental Figures_Revised Resubmission_Eric Laing.docx

## Data Availability

Data and key materials utilized in this study are available as a sharable resource upon reasonable request to the corresponding authors.

## Authors’ information

MSR and LY are employees of the Henry M. Jackson Foundation for the Advancement of Military Medicine. EDL is a federal employee within the Department of Defense. The opinions and assertions expressed herein are those of the authors and do not reflect the official policy or position of the U.S. Department of Defense, the Uniformed Services University of Health Sciences, Henry M. Jackson Foundation for the Advancement of Military Medicine, or any other agency of the U.S Government. Certain commercial equipment, instruments, software, or materials may be identified in this paper in order to specify the experimental procedure adequately. Such identification is not intended to imply recommendation or endorsement by the U.S. Department of Defense, the Uniformed Services University of Health Sciences, or Henry M. Jackson Foundation for the Advancement of Military Medicine, nor is it intended to imply that the materials or equipment identified are necessarily the best available for the purpose.
