# Supplementary material for: Seroprevalence of Nipah virus and related paramyxoviruses in native frugivorous bats, Luzon, Philippines
Source: Emerg Microbes Infect. 2025 Sep 29;14(1):2555720. doi: 10.1080/22221751.2025.2555720 (PMC12481536; doi:10.1080/22221751.2025.2555720)
Supplement: Supplemental Figures_Revised Resubmission_Eric Laing.docx [file TEMI_A_2555720_SM3187.docx]

**Supplemental Figures**

STable 1. Total number of bats collected by species and location.

|  |  | **Location** | | | | | **Sex** | | **Age*** | | | | |
| --- | --- | --- | --- | --- | --- | --- | --- | --- | --- | --- | --- | --- | --- |
| **Bat species** | **Total N** | **Infanta** | **Agno** | **DRT** | **Burdeos** | **Tayabas** | **M** | **F** | **Neo** | **SbAd** | **Juv** | **YgAd** | **Ad** |
| Flying Foxes (*P. vampyrus, P. hypomelanus, A. jubatus)* | 654 | 380 | 268 | 0 | 6 | 0 | 366 | 288 | 2 | 121 | 52 | 91 | 388 |
| *Rousettus amplexicaudatus* | 3147 | 0 | 189 | 1157 | 1137 | 664 | 1293 | 1854 | 3 | 843 | 763 | 549 | 989 |
| *Eoncyteris* species (*E. spelaea* & *robusta*) | 287 | 0 | 91 | 81 | 104 | 11 | 166 | 121 | 0 | 81 | 51 | 53 | 102 |
| *Cynopterus*  *luzoniensis* | 872 | 0 | 714 | 2 | 4 | 152 | 391 | 481 | 0 | 207 | 83 | 106 | 476 |
| *Ptenochirus*  *jagori* | 214 | 0 | 46 | 6 | 1 | 161 | 84 | 130 | 0 | 64 | 6 | 23 | 127 |

*Ages categories are neonates (neo), subadults (SbAd), juveniles (Juv), young adults (YgAd), and adults (Ad).

**
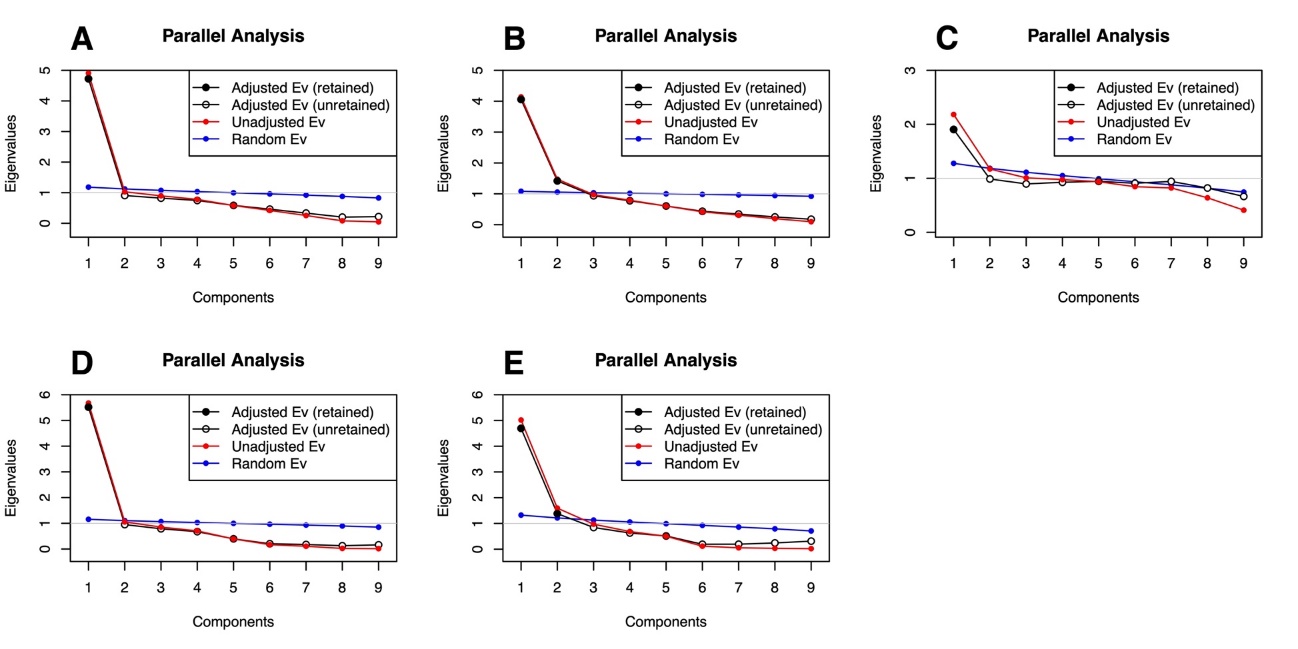
**

SFigure 1. Parallel analysis of Scree plots for each bat species group.

Parallel analysis graphs of eigenvalues associated with each principal components for A) Flying foxes (*P. hypomelanus*, *P. vampyrus*, and *A. jubatus*), B) *R. amplexicaudatus*, C) *E.* species (*E. robusta* and *spelaea*), D) *C. luzoniensis*, and E) *P. jagori*, comparing calculated eigenvalues to stimulated eigenvalues. For each plot, the plot “elbow” is located at component 2, which is the minimum number of components to include and account for most of the variance.

STable 2. Summary of eigenvalues, variance (%), and cumulative variance (%) for principal component analysis of each bat species antibody results.

|  | Flying Foxes* | | | *R. amplexicaudatus* | | | *E. species*** | | | *C. luzonensis* | | | *P. jagori* | | |
| --- | --- | --- | --- | --- | --- | --- | --- | --- | --- | --- | --- | --- | --- | --- | --- |
|  | Eigenvalue | Variance (%) | Cumulative variance (%) | Eigenvalue | Variance (%) | Cumulative variance (%) | Eigenvalue | Variance (%) | Cumulative variance (%) | Eigenvalue | Variance (%) | Cumulative variance (%) | Eigenvalue | Variance (%) | Cumulative variance (%) |
| Dim 1 | 4.91 | 54.52 | 54.52 | 4.14 | 45.99 | 45.99 | 2.18 | 24.25 | 24.25 | 5.68 | 63.06 | 63.06 | 5.02 | 55.75 | 55.75 |
| Dim 2 | 1.03 | 11.44 | 65.96 | 1.47 | 16.38 | 62.37 | 1.17 | 13.04 | 37.28 | 1.06 | 11.73 | 74.79 | 1.59 | 17.72 | 73.47 |
| Dim 3 | 0.90 | 9.97 | 75.93 | 0.97 | 10.79 | 73.17 | 1.01 | 11.22 | 48.50 | 0.85 | 9.47 | 84.26 | 0.97 | 10.81 | 84.28 |
| Dim 4 | 0.78 | 8.68 | 84.61 | 0.79 | 8.80 | 81.96 | 0.98 | 10.86 | 59.36 | 0.71 | 7.85 | 92.11 | 0.69 | 7.62 | 91.89 |
| Dim 5 | 0.58 | 6.46 | 91.08 | 0.60 | 6.72 | 88.68 | 0.94 | 10.42 | 69.78 | 0.39 | 4.38 | 96.49 | 0.50 | 5.60 | 97.49 |
| Dim 6 | 0.42 | 4.68 | 95.75 | 0.42 | 4.63 | 93.32 | 0.85 | 9.40 | 79.17 | 0.17 | 1.90 | 98.39 | 0.12 | 1.30 | 98.79 |
| Dim 7 | 0.25 | 2.82 | 98.58 | 0.31 | 3.44 | 96.76 | 0.82 | 9.14 | 88.32 | 0.11 | 1.20 | 99.59 | 0.05 | 0.61 | 99.40 |
| Dim 8 | 0.08 | 0.90 | 99.48 | 0.20 | 2.17 | 98.93 | 0.64 | 7.11 | 95.43 | 0.02 | 0.26 | 99.84 | 0.03 | 0.37 | 99.77 |
| Dim 9 | 0.05 | 0.52 | 100.00 | 0.10 | 1.07 | 100.00 | 0.41 | 4.57 | 100.00 | 0.01 | 0.16 | 100.00 | 0.02 | 0.23 | 100.00 |

*includes *P. hypomelanus*, *P. vampyrus*, and *A. jubatus*

**includes *E. robusta* and *E. spelaea*


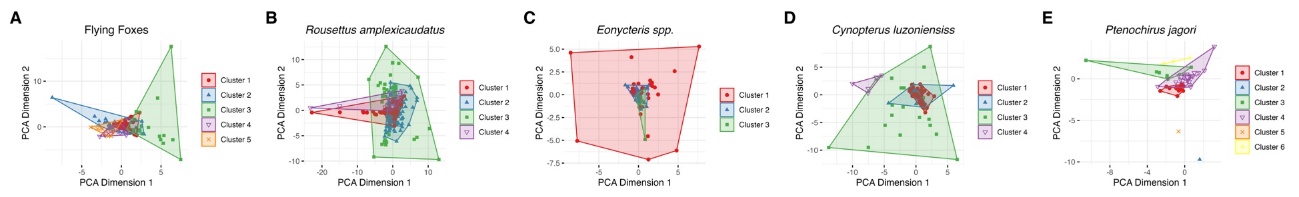


SFigure 2. PCA scatterplots with k-medoid cluster of multiple bat species group for multiplex paramyxovirus serology.

Scatterplots displaying serology results to eight paramyxovirus G and HN antigens and a mock control protein for Philippines frugivorous bats after analysis via principal component analysis (PCA), where six principal components were retained for >70% cumulative explained variance across all bat species groups. Results are visualized relative to the first two principal components (Component 1 & 2) and are shaded according to cluster assignments after k-medoids clustering. The visual overlapping of clusters appears because the full depth of separation among clusters is not fully observable with a two-dimensional visual representation. Similarly, these clusters are centered about the medoid data point for each cluster, which may not be obviously observed when visualized by the primary component axes. Bat species groups include A) flying foxes (*P. vampyrus, P. hypomelanus, A. jubatus*; N = 654), B) *R. amplexicaudatus*; N = 3147, C) *Eonycteris* species (*E. spelaea* & *E. robusta*; N = 287), D) *C. luzoniensis* (N = 872), and E) *P. jagori* (N = 214) that were collected monthly on Luzon island from July 2023 - August 2024, excluding August 2023.

**
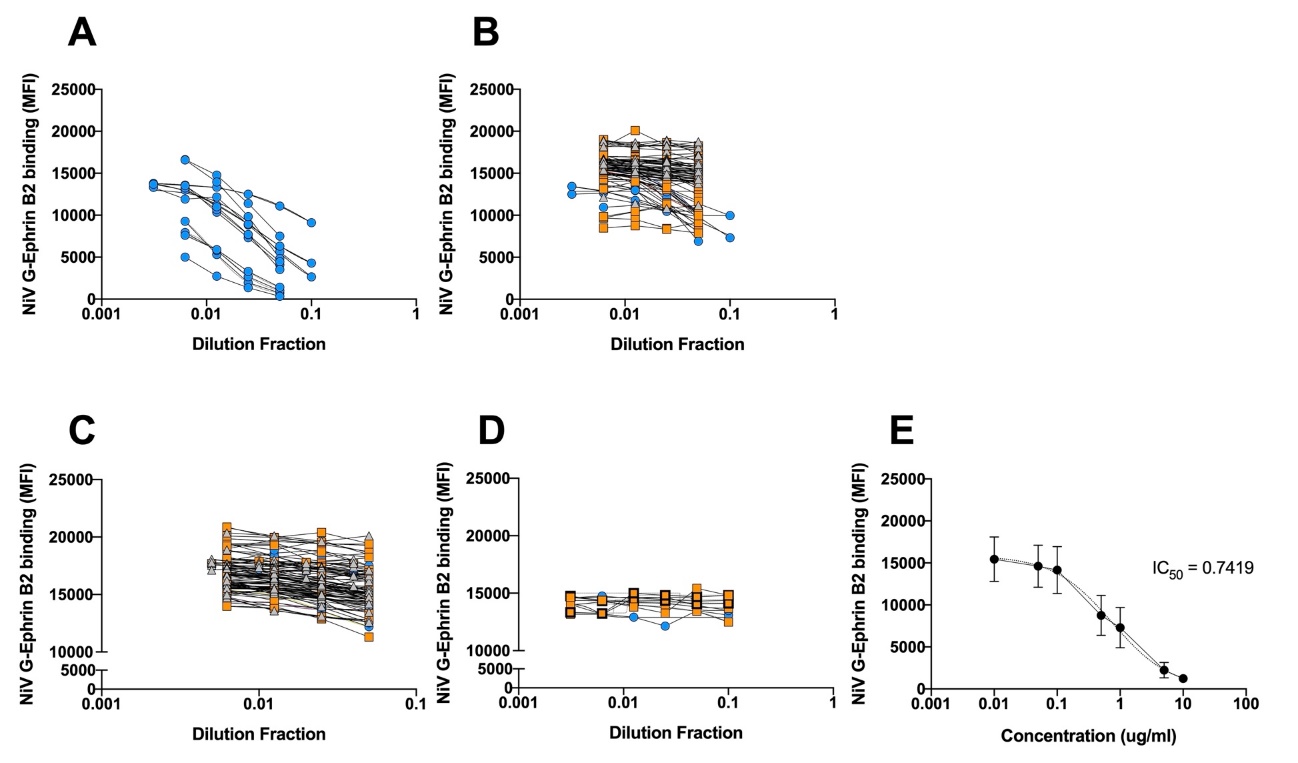
**

SFigure 3. Neutralization curves from surrogate NiV virus neutralization test (sVNT) for multiple bat species.

Neutralizing activity of selected bat sera samples for NiV, assessed with an inhibition vs. response four-parameter nonlinear fit analysis. Bat samples were selected based on NiV binding MFI values, with bin 1 from 500-1000 (gray triangle), bin 2 from 1000-3000 (orange square), and bin 3 being >3000 (blue circle) MFI; serum samples from flying foxes were included in bin 1 (N = 35), bin 2 (N= 30), and bin 3 (N= 17). A) Flying fox samples that formed proper sigmoidal curves, from which an IC50 could be calculated (N = 12), B) flying fox samples that did not form proper sigmoidal curves (N = 70), C) *R. amplexicaudatus* samples (N = 84), D) C*. luzoniensis* (N = 6) and *P. jagori* (N= 2, bolded points) samples, and (E) Anti-NiV monoclonal antibody control (N = 13) used as a positive control for formation of sigmoidal curves.

**
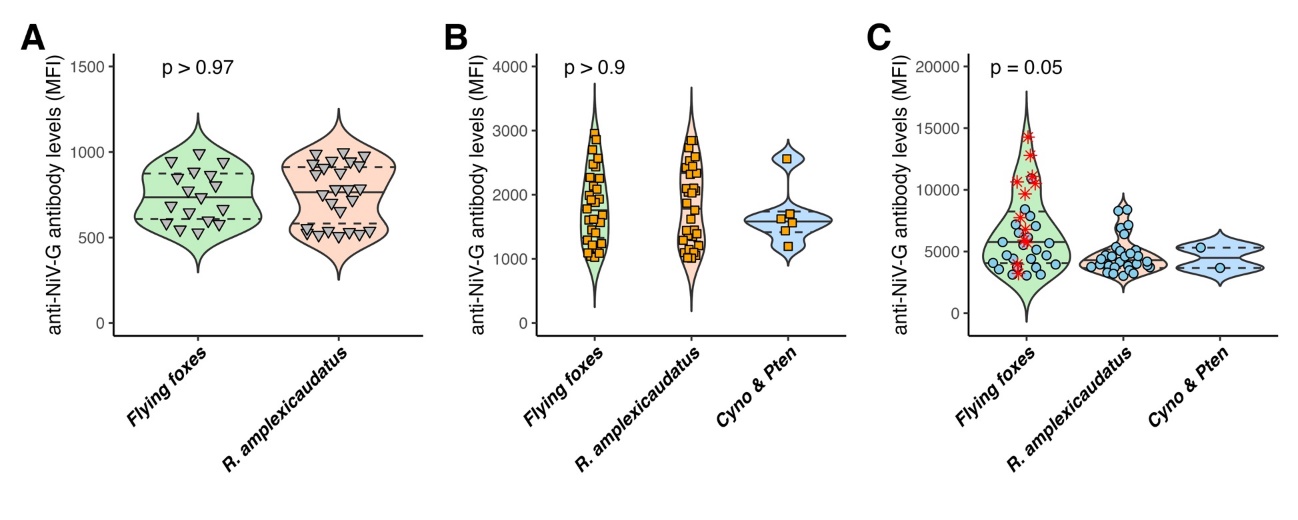
**

SFigure 4. Comparison of bat samples selected to examine NiV neutralization capacity in flying foxes, *R. amplexicaudatus*, and *C. luzoniensis* and *P. jagori* bats.

Comparison of anti-NiV antibody levels for bat samples selected for testing NiV neutralizing capacity at varying anti-NiV binding MFI ranges to ensure matching of comparable samples. A) Comparison of flying fox (N = 17) and *R. amplexicaudatus* (N = 25) samples selected at 500-1000 MFI (Bin 1, gray triangle) with no significant differences (p>0.05). B) Comparison of flying fox (N = 30), *R. amplexicaudatus* (N = 30), and *C. luzoniensis* and *P. jagori* bats (N = 6) samples selected at 1000-3000 MFI (Bin 2, orange square), with no significant differences (p>0.05). C) Comparison of flying fox (N = 35), *R. amplexicaudatus* (N = 29), and *C. luzoniensis* and *P. jagori* bats (N = 2) samples selected at >3000 MFI (Bin 3, blue circle). Flying foxes and *R. amplexicaudatus* bats at bin 3 were significantly different (p<0.05). All other comparisons were non-significant (p>0.05). Red stars indicate samples that had detectable NiV neutralization activity.

**
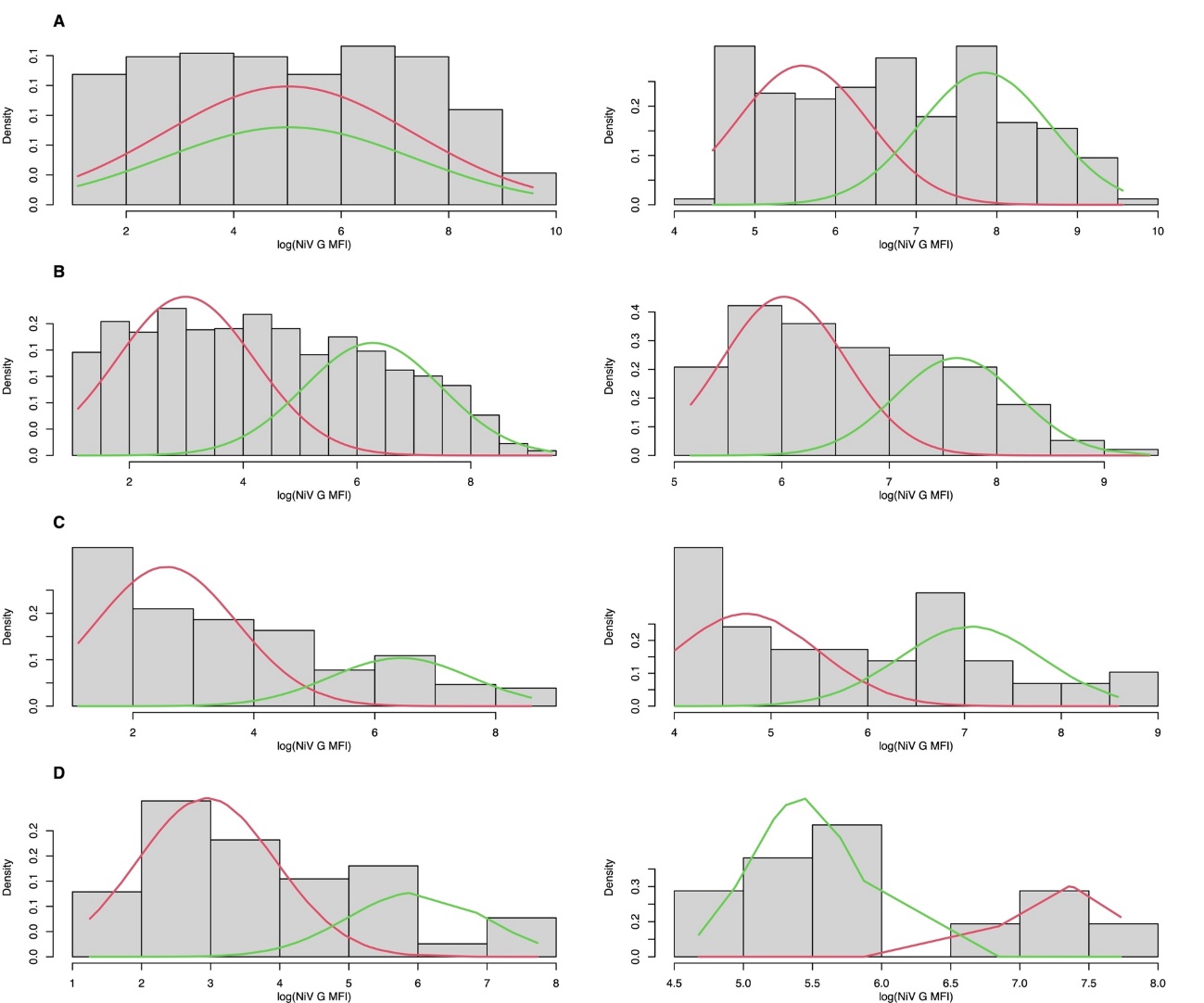
**

SFigure 5. Two population-univariate mixture model density plots of the NiV serological responses for multiple bat groups for seropositivity cutoff determination.

Mixture model density plots of log-transformed anti-NiV antibody responses (MFI) of A) flying foxes (*P. vampyrus*, *P. hypomelanus*, *A. jubatus*), B) *R. amplexicaudatus*, C) *C. luzoniensis*, and D) *P. jagori*. The first column is a two population-univariate mixture model analysis on all data and the second column is analysis on data excluding values that fall within assay noise MFI. Assay noise was determined by 95% specificity of mock MFI specific to each species.

STable 3. Calculated NiV seropositivity cutoffs at 90% sensitivity for a two populations mixture model analysis of selected bat species groups under conditions of retaining and excluding data within assay noise.

| **Bat Type** | **Total N** | **Virus** | **Seropositivity cutoff**  **(MFI; % specificity)** | **Seropositivity cutoff, noise removed**  **(MFI; % specificity)** | **Assay noise threshold**  **(mock MFI, 95% specificity)** |
| --- | --- | --- | --- | --- | --- |
| Flying foxes (*P. vampyrus*, *P. hypomelanus*, *A. jubatus*) | 625 | NiV | 198 (95) | 904 (93) | 87 |
| *R. amplexicaudatus* | 3105 |  | 113 (93) | 984 (93) | 172 |
| *C. luzoniensis* | 870 |  | 140 (98) | 455 (97) | 53 |
| *P. jagori* | 214 |  | 110 (96) | 947 (100) | 84 |


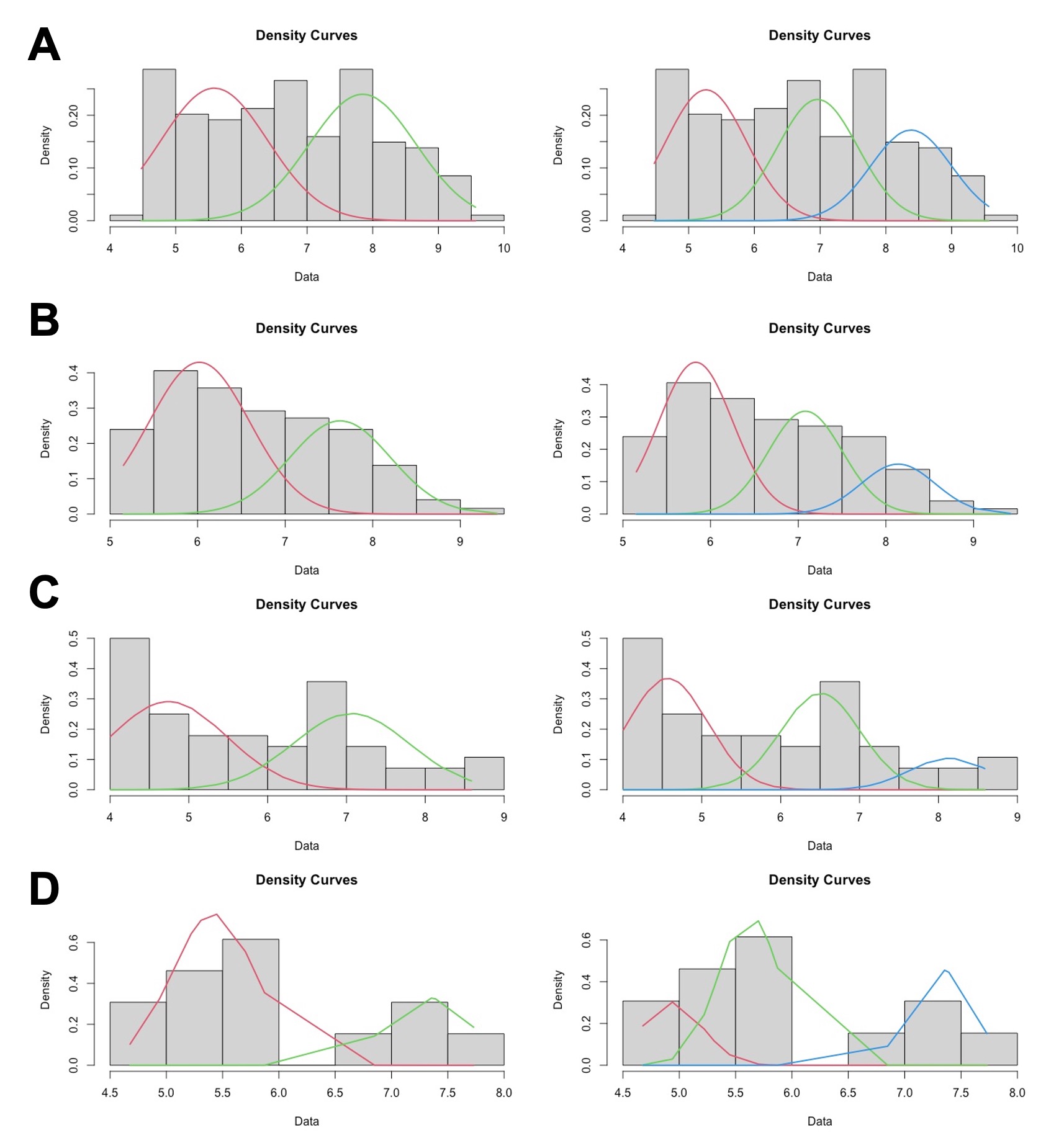


SFigure 6. Two vs. three population-univariate mixture model density plots of the NiV serological responses for multiple bat groups for seropositivity cutoff determination.

Mixture model density plots of log-transformed anti-NiV antibody responses (MFI) of A) flying foxes (*P. vampyrus, P. hypomelanus, A. jubatus*), B) *R. amplexicaudatu*s, C) *C. luzoniensis*, and D) *P. jagori*, excluding values that fall within assay noise MFI (determined by 95% specificity of mock MFI values specific to each bat type). The first column is univariate mixture model analysis set to two populations, and the second column is analysis set to three populations.

**STable 4.** Seropositivity cutoffs for NiV specific to each bat type based on univariate mixture model analysis for two and three population assumptions to calculate cutoffs with 90% sensitivity.

| **Bat Type** | **Seropositivity cutoff 2 populations (MFI, % specificity)** | **Seropositivity cutoff 3 populations (MFI, % specificity)** |
| --- | --- | --- |
| Flying foxes (*P. vampyrus, P. hypomelanus, A. jubatus*) | 904 (93) | 481 (93)  1993 (85) |
| *R. amplexicaudatus* | 984 (93) | 691 (95)  1992 (89) |
| *C. luzoniensis* | 455 (97) | 349 (99)  1786 (97) |
| *P. jagori* | 947 (99) | 195 (89)  1082 (99) |


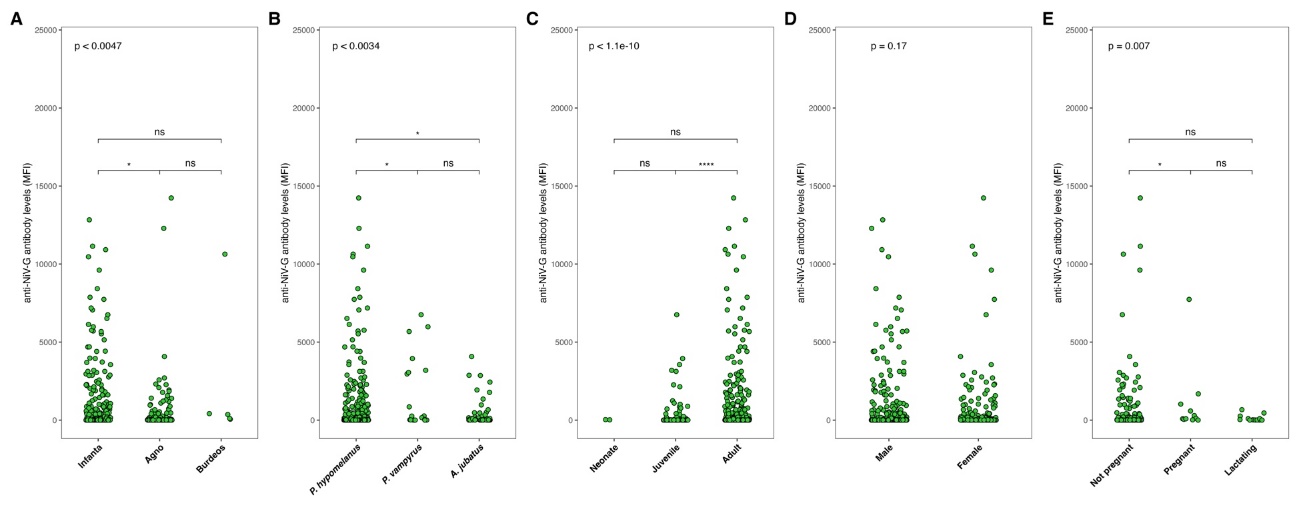


SFigure 7. Stratification of flying fox anti-NiV antibody levels by major demographic factors.

Comparisons of flying fox anti-NiV antibody levels (MFI) stratified by major demographic factors and assessed using Kruskal-Wallis tests for greater than two comparisons (Dunn’s test multiple comparisons) and Wilcoxon test for two comparisons. A) Location comparison of flying fox sera samples collected in Infanta (N = 380), Agno (N = 268), and Burdeos (N = 6), with significant differences between Agno and Infanta (p<0.05). B) Flying fox species comparison of *P. hypomelanus* (N = 558), *P. vampyrus* (N = 21), and *A. jubatus* (N = 70), with significant differences between *P. hypomelanus* and *P. vampyrus*, and *P. hypomelanus* and *A. jubatus* (p<0.05). C) Age comparison of neonate (N = 2), juvenile (N = 173), and adult (N = 479) flying foxes, with significant differences between juveniles and adults (p<0.0001). D) Comparison of male (N = 366) and female (N = 288) flying foxes, with no significant differences observed. E) Comparison of female flying fox reproductive status of not pregnant (N = 261), pregnant (N = 12), and lactating (N = 15), with significant differences observed between not pregnant and pregnant (p<0.05).
